# Robotic vs. Laparoscopic Adrenalectomy for Pheochromocytoma—A Systematic Review and Meta-Analysis

**DOI:** 10.3390/jcm14113806

**Published:** 2025-05-29

**Authors:** Alessio Giordano, Andrea Balla, Paolo Prosperi, Salvador Morales-Conde, Carlo Bergamini

**Affiliations:** 1 Emergency Surgery Unit, Department of Emergency and Acceptance, Careggi University Hospital, 50134 Florence, Italy; prosperip@aou-careggi.toscana.it (P.P.); drcarlobergamini@gmail.com (C.B.); 2 Department of General and Digestive Surgery, University Hospital Virgen Macarena, University of Sevilla, 41009 Sevilla, Spain; andrea.balla@gmail.com (A.B.); smoralesc@gmail.com (S.M.-C.)

**Keywords:** adrenal surgery, robotic surgery, pheochromocytoma, laparoscopic surgery

## Abstract

**Background:** The application of robotic adrenalectomy (RA) has been increasing. However, there is still controversy about whether RA is more feasible than laparoscopic adrenalectomy (LA) for pheochromocytoma (PHEO). **Methods:** We conducted a systematic review of published articles between 2013 and 2025 according to the PRISMA statement and the Cochrane Handbook for systematic reviews of interventions. The search was conducted in MEDLINE (PubMed, Scholar, and Cochrane databases). **Results**: Overall, seven studies including 879 patients (RA 358; LA 521) were included. RA might have larger tumor size (MD −0.66, 95% CI −1.18 to 0.13; *p* < 0.00001) but not for BMI patients (MD −0.24, 95% CI −1.44 to 0.96; *p* < 0.00001). There were no statistically significant differences in intraoperative complication, conversion to open surgery, postoperative complications, transfusion rate, and perioperative hemodynamic outcomes with the exception of a higher lowest systolic blood pressure in the LA group (MD −1.09, 95% CI −2.35 to 0.18; *p* < 0.00001). Moreover, estimated blood loss (MD 29.52, 95% CI 4.19 to 54.84; *p* < 0.00001), operative time (MD 3.85, 95% CI −16.11 to 23.80; *p* < 0.00001), and the length of hospital stay were in favor of RA (MD 0.42, 95% CI 0.09 to 0.74; *p* < 0.0001). **Conclusions**: Both LA and RA are safe and feasible approaches for adrenalectomy in the case of pheochromocytoma. RA seems to have better perioperative results, but further prospective randomized control studies are required to draw definitive conclusions.

## 1. Introduction

Pheochromocytoma (PHEO) is a neuroendocrine tumor arising from the chromaffin cells of the adrenal medulla [1]. These tumors often secrete catecholamines—epinephrine, norepinephrine, and dopamine—leading to clinical manifestations such as headache, palpitations, and excessive sweating [1,2,3,4]. Despite being historically described as a classical triad, these symptoms occur in only about 25% of cases [2,3]. Hypertension remains the most frequent presentation, reported in 80–90% of patients [2], and approximately 12% of patients may develop cardiovascular complications, particularly in those with larger tumors at diagnosis [5].

Surgical resection through adrenalectomy is considered the gold standard for treating PHEO [5,6]. Traditionally, open adrenalectomy was the preferred method, although it is often associated with significant morbidity due to the extensive incisions and tissue dissection required [6,7,8]. Since the 1990s, laparoscopic adrenalectomy (LA) has emerged as a less invasive alternative, offering comparable efficacy [6]. Meta-analyses have shown that LA results in reduced intraoperative blood loss, lower rates of hemodynamic instability, and better postoperative recovery compared to the open approach [7,8].

Selecting the most appropriate surgical technique is a key component in the management of PHEO. Various laparoscopic strategies have been described, with the anterior and lateral transperitoneal approaches, as well as the lateral and posterior retroperitoneal routes, being among the most frequently utilized [9]. The lateral transperitoneal approach is particularly favored for its enhanced visualization facilitated by gravitational organ retraction, its alignment with standard anatomical landmarks, and its suitability for resecting larger adrenal masses [4].

In patients with prior abdominal surgeries, the posterior retroperitoneal approach offers the advantage of avoiding intra-abdominal adhesions, enabling direct access to the adrenal gland without manipulation of other abdominal organs. This technique also allows for bilateral adrenalectomy without repositioning the patient [10].

Both transperitoneal and retroperitoneal techniques are considered safe and effective; however, subtle distinctions exist, and several factors—including the surgeon’s experience, the patient’s body habitus, comorbidities, and potential for complications—should guide the choice of approach.

Laparoscopy, despite its advantages, presents inherent technical limitations such as restricted instrument motion, two-dimensional imaging, and potential amplification of physiologic tremors, which can complicate procedures involving large PHEOs [11]. Robotic adrenalectomy (RA) has been developed to address these challenges, offering enhanced dexterity, tremor filtration, and three-dimensional visualization [12]. RA has increasingly been reported as a safe and feasible alternative for PHEO surgery [11,12,13], although some studies, such as that by Park et al., have not demonstrated significant clinical advantages over the laparoscopic technique [14].

There are concerns that the robotic approach may lead to increased cardiovascular risk, potentially due to the absence of haptic feedback, which could contribute to excessive catecholamine release during manipulation. Nonetheless, these findings are based on small sample sizes, limiting the strength of the conclusions [11,12]. Thus, the question of whether LA or RA offers superior outcomes in the treatment of PHEO remains unresolved.

In light of these considerations, we conducted a systematic review and meta-analysis to compare laparoscopic and robotic adrenalectomy for PHEO, focusing on intraoperative metrics and 30-day postoperative outcomes.

## 2. Material and Methods

Institutional review board approval and informed consent from participants were unnecessary for the present study.

### 2.1. Search Strategy

We conducted a systematic review of published articles between 2013 and 2025 according to the Preferred Reporting Items for Systematic Review and Meta-Analysis (PRISMA) statement [15] and according to the Cochrane Handbook for systematic reviews of interventions [16]. The review protocol was not registered. The search was conducted in MEDLINE (PubMed, Scholar, and Cochrane databases) [17] using the following string research: (Pheocromocitoma) AND (surgery), OR (adrenalectomy), OR (laparoscopy), OR (mini-invasive), OR (mininvasive), OR (robot).

Cross-referencing using articles initially identified was also performed to include additional articles that did not explicitly focus on the topic but reported the requested information in subgroup analyses. Two reviewers (C.B. and A.G.) evaluated the articles retrieved from searches, and disagreements on study selection or data extraction were resolved by consensus and discussion among reviewers.

The PICOS (Population, Intervention, Comparison, Outcomes, and Studies) question was generated from a discussion within the authors. The following PICOS question was adopted:

P: Patients with a pheochromocytoma.

I: Laparoscopic adrenalectomy.

C: Robotic adrenalectomy.

O: Intraoperative complications, blood loss, hemodynamic instability, conversion, operative time, postoperative complications, length of hospital stay (LOS), mortality.

S: Randomized controlled trials, non-randomized controlled trials (retrospective and prospective cohort studies)

### 2.2. Inclusion Criteria

Articles written in English, Italian, and Spanish were included. All articles included patients who underwent minimally invasive (laparoscopic or robotic) adrenalectomy for PHEO.

### 2.3. Exclusion Criteria

Articles were excluded if they included patients with other types of adrenal lesions; if they included a cohort of patients retrieved from an article already included; if it was not possible to isolate data only regarding adrenalectomy for PHEO; if they were comments, case reports, correspondence and letters to the editor, editorials, technical surgical notes, conference articles, imaging studies, and articles involving animals.

### 2.4. Risk of Bias Assessment of Included Articles

Three authors (C.B., A.G., A.B.) used the Risk Of Bias In Non-randomised Studies—of Interventions (ROBINS-I) tool and Cochrane Risk of Bias 2.0 (RoB 2.0) tool in non-randomized and randomized trials, respectively, to assess the risk of bias of the included studies [18,19].

### 2.5. Study Design

After screening the title and abstract, articles that fulfilled the inclusion criteria were identified, and their full text was reviewed. Data were extracted and stored in an Excel chart (Microsoft Corporation, Redmond, WA, USA).

The following data were extracted from each article: first author, year of publication, type of study, number of patients, sex, age, body mass index (BMI), lesion side (right, left, bilateral), lesion size, American Society of Anesthesiologists (ASA) grade, minimally invasive approach (laparoscopic or robotic), intraoperative events/complications, hemodynamic instability, estimated blood loss (EBL), intraoperative systolic blood pressure and heart rate, conversion to open surgery, operative time, postoperative complications, LOS, and mortality.

### 2.6. Statistical Analysis

Categorical data were reported as absolute numbers and percentages, while continuous variables were presented as means with corresponding standard deviations (SD). Variables were included in the pooled analysis only when reported by at least two independent studies. Statistical analyses were conducted using Review Manager software (RevMan version 5.4.1; The Nordic Cochrane Centre, Cochrane Collaboration, www.training.cochrane.org, accessed on 1 March 2025). Two independent reviewers (A.B. and A.G.) verified the data entries in the forest plot tables to ensure accuracy.

For dichotomous outcomes, relative risks (RRs) with 95% confidence intervals (CIs) were calculated, while mean differences (MDs) with 95% CIs were used for continuous variables. When studies reported continuous data as medians and ranges, the corresponding means and SDs were estimated using the method described by Hozo et al. [20]. An RR was considered statistically significant at a *p* value < 0.05 if the 95% CI did not include 1, and an MD was deemed significant under the same P threshold if the 95% CI did not include 0.

Statistical heterogeneity across studies was assessed using both the χ^2^ test and Higgins’ I^2^ statistic. A χ^2^ *p* value < 0.100 combined with an I^2^ value > 50% was interpreted as indicating substantial heterogeneity. In addition to statistical heterogeneity, clinical (e.g., differences in baseline patient characteristics, interventions, or outcome definitions) and methodological heterogeneity (e.g., variations in study design and risk of bias) were also taken into account when selecting the appropriate analytical model. Given the considerable heterogeneity observed, a random-effect model was applied to all meta-analyses [21].

### 2.7. Grading the Quality of Evidence

Three authors (C.B., A.G., A.B.) independently evaluated the quality of evidence for imprecision, inconsistency, indirectness, and publication bias according to the Grading of Recommendations, Assessment, Development, and Evaluations (GRADE) approach [22]. Certainty of evidence (CoE) was classified as very low, low, moderate, or high [22]. Subsequently, a summary table was created using the GRADE profiler software (version 3.6.1) [22].

## 3. Results

**Figure 1:**
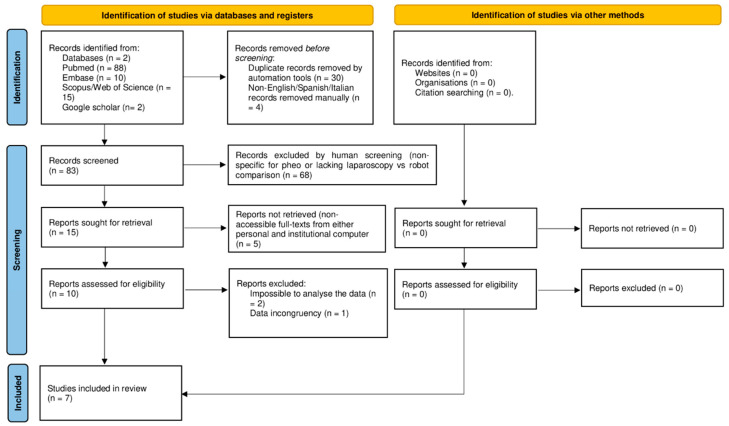
Preferred reporting items for systematic review and meta-analysis (PRISMA) flow diagram [15].

Following the inclusion and exclusion criteria, seven articles published between December 2013 and March 2025 were included [23,24,25,26,27,28,29], as shown in the PRISMA flow diagram (Figure 1) [15]. The assessment of the risk of bias based on the ROBINS-I and RoB 2.0 of the articles is shown in Table 1. Six articles included were retrospective analyses [23,25,26,27,28,29], and one article was a randomized control trial [24]. Six were single-center studies [23,24,25,27,28,29], and one was a multicenter study [26].

Table 2 shows preoperative patients’ characteristics. Overall, 879 patients were included in the analysis, of which 521 underwent LA (250 men, 48%, and 271 women, 52%), and 358 underwent RA (155 men, 43.8%, and 203 women, 56.7%). In the LA group, 265 (50.9%), 246 (47.2%), and 10 (1.9%) patients underwent right, left, and bilateral adrenalectomy, respectively. In the RA group, 179 (50%), 178 (49.7%), and 1 (0.3%) patients underwent right, left, and bilateral adrenalectomy, respectively. In the LA group, mean lesion size ranged between 4 and 7.6 cm, while in the RA group, it ranged between 4.6 and 8 cm. ASA grade was I–II and III–IV in 128 (39.8% out of 322 patients) and 194 (60.2% out of 322 patients) patients of the LA group, respectively, and 90 (43.9% out of 205 patients) and 115 (56.1% out of 205 patients) in the RA group, respectively [21,22,23,24,25,26,27].

Table 3 reports perioperative results. In the LA group, 175 (40.3%) and 259 (59.7%) adrenalectomies out of 434 were performed by transperitoneal and retroperitoneal approaches, respectively. Meanwhile, in the RA group, 165 (51.9%) and 153 (48.1%) adrenalectomies out of 318 were performed by transperitoneal and retroperitoneal approaches, respectively [21,22,23,24,25,26,27].

Intraoperatively, in the LA group, 147 events (28.2%) occurred, including cardiac arrhythmia (1, 0.2%), transfusions (25, 4.8%), hemodynamic instability (113, 21.7%), and hypertensive crisis (8, 1.5%), and 6 complications (1.2%), including spleen bleeding requiring open splenectomy (1, 0.2%), organ injury requiring suture (2, 0.4%), cava vein injury requiring suture (1, 0.2%), bleeding (1, 0.2%), and suspecting spleen rupture requiring open exploration (1, 0.2%). In the RA group, 56 events (15.6%) occurred, including transfusions (19, 5.3%), hemodynamic instability (34, 9.5%), ventricular fibrillation (1, 0.3%), and hypertensive crisis (2, 0.6%), and 3 complications (0.8%) including organ injury requiring suture (1, 0.3%), bleeding (1, 0.3%), and diaphragmatic rupture (1, 0.3%) [23,24,25,26,27,28,29].

Conversion to open surgery occurred in 13 (2.5%) and 2 (0.6%) patients in the LA and RA groups, respectively. The reasons for conversion to open surgery in the LA group were as follows: tumor bleeding (1, 0.2%), accessory renal vein bleeding (1, 0.2%), difficult dissection (4, 0.8%), inadequate surgical field exposure (2, 0.4%), spleen bleeding (1, 0.2%), and four not reported. Meanwhile, in the RA group, the reasons for conversion were tumor adhesions (1, 0.3%) and inadequate surgical field exposure (1, 0.3%) [23,24,25,26,27,28,29].

Postoperative complications and transfusions occurred in 88 (16.9%) and 42 patients (11.7%) and 16 (3.1%) and 17 (4.7%) patients, in the LA and RA groups, respectively. Mortality occurred in one patient in the LA group (0.2%) [23,24,25,26,27,28,29].

### Meta-Analysis

**Figure 2:**
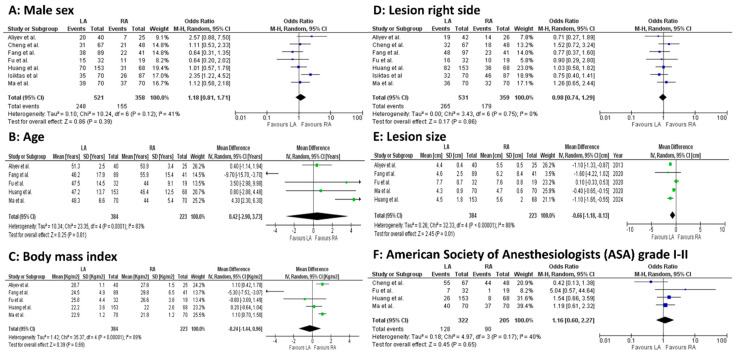
Meta-analysis of preoperative outcomes. (**A**): Male sex. (**B**): Age. (**C**): Body mass index. (**D**): Lesion right side. (**E**): Lesion size. (**F**): American Society of Anesthesiologists (ASA) grade I–II [23,24,25,26,27,28,29].

Before-surgery age (5 studies, 607 patients; MD 0.42, 95 percent CI −2.90 to 3.73; *p* = 0.0001; I^2^ = 83%, random-effect model), BMI (5 studies, 607 patients; MD −0.24, 95 percent CI −1.44 to 0.96; *p* < 0.00001; I^2^ = 89%, random-effect model), and lesion size (5 studies, 607 patients; MD −0.66, 95 percent CI −1.18 to 0.13; *p* < 0.00001; I^2^ = 88%, random-effect model) were statistically significantly different between the LA and RA groups (Figure 2A–F).

**Figure 3:**
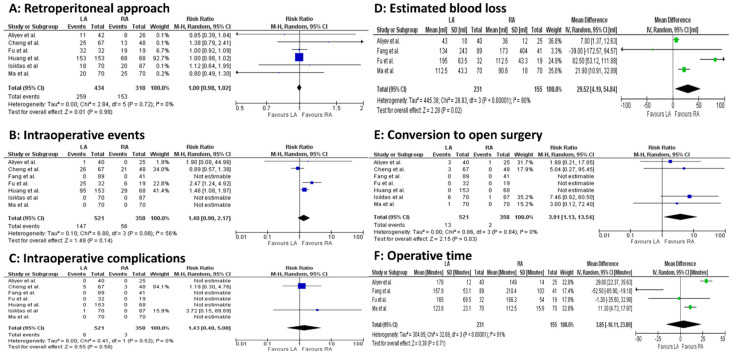
Meta-analysis of intraoperative outcomes. (**A**): Retroperitoneal approach. (**B**): Intraoperative events. (**C**): Intraoperative complications. (**D**): Estimated blood loss. (**E**): Conversion to open surgery. (**F**): Operative time [23,24,25,26,27,28,29].

Intraoperatively, statistically significant differences occurred for EBL (4 studies, 386 patients; MD 29.52, 95 percent CI 4.19 to 54.84; *p* < 0.00001; I^2^ = 90%, random-effect model; CoE: Low) and operative time (4 studies, 386 patients; MD 3.85, 95 percent CI −16.11 to 23.80; *p* < 0.00001; I^2^ = 91%, random-effect model; CoE: Low), both in favor of the LA group (Figure 3A–F).

**Figure 4:**
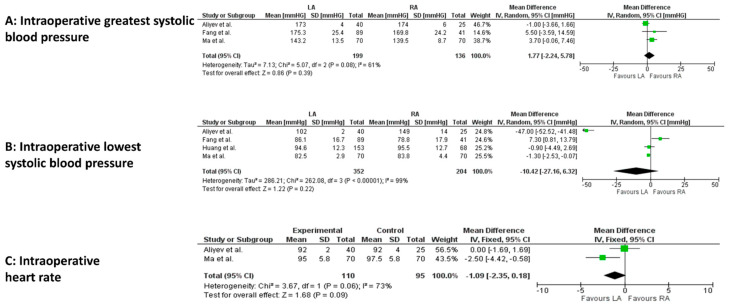
Meta-analysis of intraoperative cardiovascular variables. (**A**): Intraoperative greatest systolic blood pressure. (**B**): Intraoperative lowest systolic blood pressure. (**C**): Intraoperative heart rate [23,24,26,29].

Among intraoperative cardiovascular variables, a statistically significant difference occurred for the lowest systolic blood pressure (4 studies, 556 patients; MD −1.09, 95 percent CI −2.35 to 0.18; *p* < 0.00001; I^2^ = 99%, random-effect model; CoE: Low) in the LA group (Figure 4A–C).

**Figure 5:**
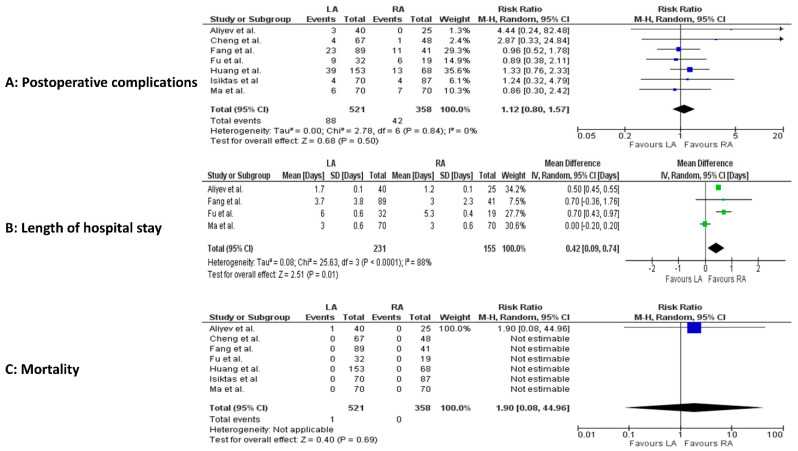
Meta-analysis of postoperative results. (**A**): Postoperative complications. (**B**): Postoperative length of hospital stay. (**C**): Postoperative mortality [23,24,25,26,27,28,29].

Postoperatively, LOS was significantly shorter in the RA group (4 studies, 386 patients; MD 0.42, 95 percent CI 0.09 to 0.74; *p* < 0.0001; I^2^ = 88%, random-effect model; CoE: Low) (Figure 5A–C).

**Figure 6:**
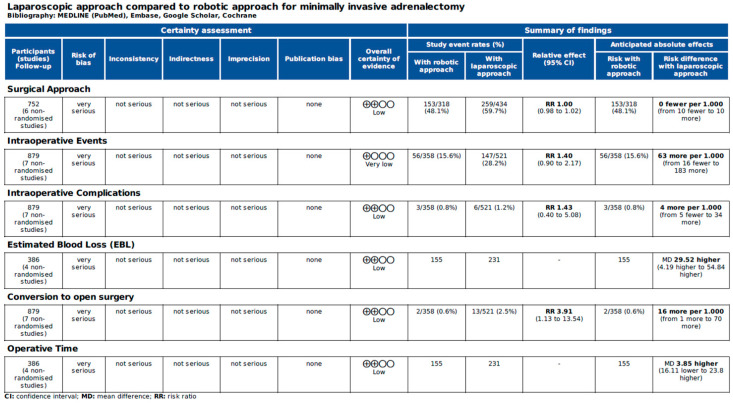
Assessment of intraoperative evidence of the included articles according to the Grading of Recommendations Assessment, Development and Evaluation (GRADE) [22].

**Figure 7:**
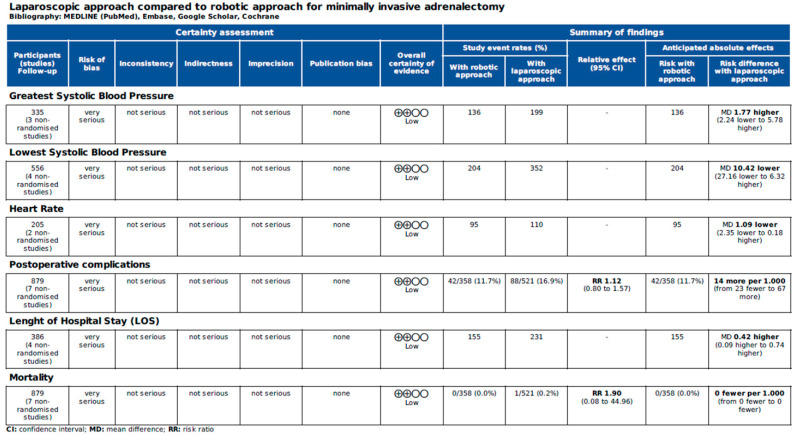
Assessment of cardiovascular variables and postoperative evidence of the included articles according to the Grading of Recommendations Assessment, Development and Evaluation (GRADE) [22].

Figure 6 and Figure 7 report the assessment of perioperative evidence according to the GRADE method of the included articles.

## 4. Discussion

Adrenalectomy can be technically demanding, particularly in the presence of large tumors, due to the retroperitoneal location of the adrenal glands and their close relationship with major vascular structures and vital abdominal organs [5,30,31]. Since its initial description by Gagner et al. in 1992 [32], the laparoscopic approach (LA) has progressively gained acceptance and is now considered the gold standard for the surgical management of most adrenal tumors [6,32]. LA has demonstrated a favorable safety profile with a low incidence of complications. Nonetheless, certain scenarios—such as large adrenal masses, the need for lymph node dissection, or surgery in obese patients—may present significant technical challenges [30,31,32,33,34]. To address the inherent limitations of laparoscopy, including two-dimensional visualization, limited instrument articulation, surgeon discomfort, and a steep learning curve, robotic-assisted surgery has been introduced. The robotic platform is being increasingly adopted in adrenal surgery, although current evidence remains limited, and large-scale comparative studies evaluating perioperative safety and short-term outcomes relative to laparoscopy are still lacking [13].

This debate in the literature is also open for what concerns the treatment of a severe such as PHEO in which surgical resection remains the therapeutic gold standard [5,6]. Therefore, we conducted a meta-analysis of the existing literature and drew objective conclusions on this topic.

In our meta-analysis, before surgery, patients in the LA group and the RA group had statistically significant differences in terms of age, BMI, and lesion size. As far as BMI is concerned, minimally invasive surgery is an improvement factor in surgical practice compared to the traditional open approach [35]. In this case, the meta-analysis shows a greater predominance of the choice of the laparoscopic approach in patients with a higher BMI, and this depends on the type of minimally invasive approach chosen with a greater prevalence of the retroperitoneal laparoscopic approach for patients with a higher BMI. The impact of obesity on outcomes after adrenalectomy for pheochromocytoma is unclear. A recent study evaluating outcomes after minimally invasive and open adrenalectomy for pheochromocytoma in obese patients, reports that obesity does not increase complications, but it does increase LOS, and the retroperitoneal approach may uniquely benefit patients with obesity [35].

The tumor size was larger in the RA group. This could be the inherent selection bias due to the operative advantages of RA [11,12,36,37,38]. Most of the studies included in this article reported large PHEOs, since the mean tumor size was larger than 4 cm or even 8 cm [23,24,25,26,27,28,29]. Large PHEOs might be related to intense vascularization, adhesion with surrounding tissues, including the inferior vena cava on the right side, and higher cancer risk, which could significantly increase the difficulty of dissection [31,39,40,41]. In general, the size does not constitute a criterion of exclusion for minimally invasive surgery because it constitutes only a predictive factor of malignancy [31,39,40,41]. Obviously, the rules of adequate and accurate manipulation to avoid the rupture of the adrenal capsule must always be followed, especially in the case of pheochromocytomas [30,39,42].

Regarding the perioperative outcomes, in the present analysis, we found a higher rate of intraoperative events (28.2% versus 15.6%), intraoperative complication (1.2% versus 0.8%), conversion to open surgery (2.5% versus 0.6%) and postoperative complications (16.9% versus 11.7%) in the LA group in comparison to the RA group. Moreover, EBL, operative time, and LOS were in favor of RA [23,24,25,26,27,28,29].

Another important aspect to consider is that pheochromocytomas are often catecholamine-secreting tumors. Intraoperatively, hemodynamic crisis is the most challenging factor during the manipulation of the tumor [43,44,45]. Due to the lack of tactile feedback, several studies were concerned about the risk of bleeding, cardiovascular accidents, and intraoperative hypertensive crisis during the dissection of PHEO [45]. Our study found similar perioperative hemodynamic outcomes between the two groups with the exception of a higher lowest systolic blood pressure. However, this could be related to the preoperative α- and β blockers, which could significantly improve perioperative hemodynamic stability [45].

This systematic review and meta-analysis study has several limitations such as the small number of articles included and, as a consequence, the small number of patients for each group, which restricts the statistical power and generalizability of the findings. Furthermore, it is not possible to carry out a sub-analysis based on lesion side, BMI, and surgical approach which could add value to the analysis, due to data reported in the included studies. Moreover, the retrospective nature of most of the included studies introduces inherent biases such as selection and confounding, which have been acknowledged in our risk of bias assessment. The assessment of the risk of bias (Table 1) and the further GRADE assessment of the individual studies (Figure 6 and Figure 7) confirm what has been said. The high degree of heterogeneity observed in the meta-analysis complicates the pooling of results and the interpretation of outcomes, as well as the lack of adequate follow-up for the analysis of long-term oncological results, the lack of a description of centers included as high or low volume, which could influence the results, and the lack of data regarding learning curve and cost analysis. However, despite these limitations, the present study provides the wider systematic review reported in the literature until now, detailing the results of laparoscopic and robotic approaches for the treatment of pheochromocytoma and comparing them, suggesting the potential advantages of the robotic approach.

## 5. Conclusions

Both laparoscopic adrenalectomy (LA) and robotic adrenalectomy (RA) have been shown to be safe and feasible options for the surgical management of pheochromocytoma. According to the existing literature, the robotic approach may offer certain advantages over laparoscopy in selected parameters, including reduced estimated blood loss, shorter operative times, decreased length of hospital stay, and potentially improved outcomes in the treatment of large pheochromocytomas. However, due to the limited number of available studies—most of which are retrospective and observational in nature—and the significant heterogeneity in patient populations and methodologies, high-quality prospective randomized controlled trials are still needed to establish definitive evidence on the superiority of one technique over the other.

## Figures and Tables

**Table 1:** Assessment of risk of bias of included articles based on Risk Of Bias In Non-randomised Studies—of Interventions (ROBINS-I) [18] and based on Cochrane Risk of Bias 2.0 (RoB 2.0) tool for randomized trials [19].

**Assessment of risk of bias based on Risk of Bias In Non-randomised Studies—of Interventions (ROBINS-I)**								
**Author, year, type of study**	**Bias due to confounding**	**Bias in selection of participants for the study**	**Bias in classification of interventions**	**Bias due to deviations from intended interventions**	**Bias due to missing data**	**Bias in measurement of outcomes**	**Bias in selection of reported result**	**Overall**
**Aliyev, 2013, retrospective** [23]	Low	Serious	Low	Low	Low	Low	Low	Serious
**Fu, 2020, retrospective** [25]	Low	Serious	Low	Low	Low	Low	Low	Serious
**Fang, 2020, retrospective** [26]	Low	Serious	Moderate	Low	Moderate	Low	Moderate	Serious
**Isiktas, 2022, retrospective** [27]	Low	Serious	Low	Low	Low	Low	Moderate	Serious
**Cheng, 2023, retrospective** [28]	Low	Serious	Low	Low	Low	Low	Moderate	Serious
**Huang, 2024, retrospective** [29]	Low	Serious	Low	Low	Low	Low	Moderate	Serious
**Assessment of risk of bias based on Cochrane Risk of Bias 2.0 (RoB 2.0) tool for randomized trials**								
**Author, year, type of study**	**Bias arising from the randomization process**	**Bias due to deviations from intended interventions**	**Bias due to missing outcome data**	**Bias in measurement of the outcome**	**Bias in selection of the reported results**		**Overall**	
**Ma, 2020, randomized control trial** [24]	Low	Low	Moderate	Low	Moderate		Moderate	

Low: low risk of bias (the study is comparable to a randomized trial). Moderate: moderate risk of bias (the study provides sound evidence for a non-randomized study but cannot be considered comparable to a randomized trial). Serious: serious risk of bias (the study has important problems).

**Table 2:** Patients’ preoperative characteristics.

Author	Number of Patients	Sex Men:Women, n (%)	Mean Age ± Standard Deviation, Years	Mean Body Mass Index ± Standard Deviation, Kg/m^2^	Lesion Side, Right:Left:Bilateral n (%)	Mean Lesion Size ± Standard Deviation, cm	American Society of Anesthesiologists Grade, n (%)
**Aliyev et al. [23]**	LA: 40	20 (50):20 (50)	51.3 ± 2.5	28.7 ± 1.1	19 (47.5):19 (47.5):2 (5)	4.4 ± 0.4	n.r.
	RA: 25	7 (28):18 (72)	50.9 ± 3.4	27.6 ± 1.5	14 (56):10 (40):1 (4)	5.5 ± 0.5	n.r.
**Ma et al. [24]**	LA: 70	39 (55.7):31 (44.3)	Median 50 (IQR 35–58)	Median 22.8 (IQR 20.8–25.1)	36 (51.4):34 (48.6)	Median 4 (IQR 3–6)	II: 40 (57.1), III: 29 (41.4), IV: 1 (1.4)
	RA: 70	37 (52.9):33 (47.1)	Median 44 (IQR 34.5–53.5)	Median 21.9 (IQR 19.6–23.9)	32 (45.7):38 (54.3)	Median 4.6 (IQR 3.8–5.6)	II: 37 (52.9), III: 31 (44.3), IV: 2 (2.9)
**Fu et al. [25]**	LA: 32	15 (46.9):17 (53.1)	47.5 ± 14.5	25.8 ± 4.4	16 (50):16 (50)	Median 7.6 (IQR 6.6–9)	I–II: 7 (21.9), III–IV: 25 (78.1)
	RA: 19	11 (57.9):8 (42.1)	44 ± 9.1	26.6 ± 3.8	10 (52.6):9 (47.4)	Median 8 (IQR 6–9)	I–II: 1 (5.3), III–IV: 18 (94.7)
**Fang et al. [26]**	LA: 89	38 (42.7):51 (57.3)	46.2 ± 17.9 SEM	24.5 ± 4.9 SEM	48 (53.9):33 (37.1):8 (9)	4.6 ± 2.5 SEM	n.r.
	RA: 41	22 (53.7):19 (46.3)	55.9 ± 15.4 SEM	29.8 ± 6.5 SEM	23 (56.1):18 (43.9)	6.2 ± 8.4 SEM	n.r.
**Isiktas et al. [27]**	LA: 70	35 (50):35 (50)	Median 51.6 (IQR 18.3)	Median 29.1 (IQR 10.4)	32 (45.7):38 (54.3)	Median 4.1 (IQR 2.3)	n.r.
	RA: 87	26 (29.9):61 (70.1)	Median 48.7 (IQR 28)	Median 28.6 (IQR 7.4)	46 (52.9):41 (47.1)	Median 3.6 (IQR 2.5)	n.r.
**Cheng et al. [28]**	LA: 67	31 (46.3):36 (53.7)	45 (range 33–60)	22.6 (range 20.9–25.4)	32 (47.8):35 (52.2)	6 (range 5.5–7)	I–II: 55 (82.1), III–IV: 12 (17.9)
	RA: 48	21 (43.8):27 (56.2)	43.5 (range 32.5–58)	23.1 (range 21.9–24.5)	18 (37.5):30 (62.5)	6 (range 5.5–7.3)	I–II: 44 (91.7), III–IV: 4 (8.3)
**Huang et al. [29]**	LA: 153	72 (47.1):81 (52.9)	47.2 ± 13.7	22.2 ± 3.6	82 (53.6):71 (46.4)	4.5 ± 1.8	I–II: 26 (17), III–IV: 127 (83)
	RA: 68	31 (45.6):37 (54.4)	46.4 ± 12.5	22 ± 2.6	36 (52.9):32 (47.1)	5.6 ± 2	I–II: 8 (11.8), III–IV: 60 (88.2)

LA: laparoscopic adrenalectomy. RA: robotic adrenalectomy. IQR: interquartile range. n.r.: not reported. SEM: standard error of mean.

**Table 3:** Perioperative results.

Author	Number of Patients	Approach, n (%)	Intraoperative Events/Complications, n (%)	Mean EBL ± SD, ml	Mean SBP ± SD, mmHg. Greatest HR, n (%)	Conversion to Open Surgery, n (%)	Mean Operative Time ± SD, Minutes	Postoperative Complications, n (%)	Mean LOS ± SD, Days	Mortality, n (%)
**Aliyev et al. [23]**	LA: 40 ^a^	Lateral transperitoneal: 31 (73.8) Posterior retroperitoneal: 11 (26.2)	Cardiac arrhythmia: 1 (2.5)	43 ± 10	Greatest: 173 ± 4 Lowest: 102 ± 2 HR: 92 ± 2	Tumor bleeding: 1 (2.5) Accessory renal vein bleeding: 1 (2.5) Difficult dissection: 1 (2.5)	178 ± 12	Cardiac arrhythmia: 2 (5) Pleural effusion: 1 (2.5)	1.7 ± 0.1	Cardiac arrhythmia: 1 (2.5)
	RA: 25 ^b^	Lateral transperitoneal: 18 (69.2) Posterior retroperitoneal: 8 (30.8)	-	36 ± 12	Greatest: 174 ± 6 Lowest: 97 ± 3 HR: 92 ± 4	Tumor adhesions: 1 (4)	149 ± 14	-	1.2 ± 0.1	-
**Ma et al. [24]**	LA: 70	Transperitoneal: 50 (71.4) Retroperitoneal: 20 (28.6)	-	Median 100 (IQR 50–200)	Greatest median: 140 (IQR 123–170) Lowest median: 80 (IQR 80–90) Median HR: 95 (IQR 85–105)	1 (1.4)	Median 122.5 (IQR 85–165)	Pneumonia: 6 (8.6) Transfusion: 2 (2.9)	Median 3 (IQR 2–4)	-
	RA: 70	Transperitoneal: 45 (64.3) Retroperitoneal: 25 (35.7)	-	Median 100 (IQR 50–112.5)	Greatest median: 139 (IQR 125–155) Lowest median: 83 (IQR 80–95) Median HR: 95 (IQR 90–110)	-	Median 107.5 (IQR 90–145)	Pneumonia: 7 (10) Transfusion: 4 (5.7)	Median 3 (IQR 2–4)	-
**Fu et al. [25]**	LA: 32	Retroperitoneal	Transfusion: 7 (21.9) Hemodynamic instability: 18 (56.2)	Median 200 (IQR 80–300)	n.r.	-	165 ± 69.5	9 (28.1)	Median 6 (IQR 5–7)	-
	RA: 19	Retroperitoneal	Transfusion: 1 (5.3) Hemodynamic instability: 5 (26.3)	Median 100 (IQR 50–200)	n.r.	-	166.3 ± 54	6 (31.6)	Median 5 (IQR 5–6)	-
**Fang et al. [26]**	LA: 89 ^c^	n.r.	-	134 ± 243 SEM	Greatest: 175.3 ± 25.4 SEM Lowest: 86.1 ± 16.7 SEM	-	157.9 ± 53.1 SEM	23 (25.8)	3.7 ± 3.8 SEM	-
	RA: 41	n.r.	-	173 ± 404 SEM	Greatest: 169.8 ± 24.2 SEM Lowest: 78.8 ± 17.9 SEM	-	210.4 ± 103 SEM	11 (26.8)	3 ± 2.3 SEM	-
**Isiktas et al. [27]**	LA: 70	Lateral transperitoneal: 52 (74.3) Posterior retroperitoneal: 18 (25.7)	Spleen bleeding requiring splenectomy: 1 (1.4)	Median 99.9 (IQR 65)	n.r.	Inadequate surgical field exposure: 2 (2.9) Spleen bleeding: 1 (1.4) Difficult dissection for adrenal mass: 3 (4.3)	Median 180.2 (IQR 22.6)	Renal failure 1 (1.4) Acute respiratory distress syndrome: 1 (1.4) Hyponatremia: 1 (1.4) Intra-abdominal abscess: 1 (1.4)	Median 2.2 (IQR 1)	-
	RA: 87	Lateral transperitoneal: 67 (77) Posterior retroperitoneal: 20 (23)	-	Median 36.3 (IQR 35)	n.r.	Inadequate surgical exposure: 1 (1.5)	Median 166.2 (IQR 60.5)	Urinary tract infection: 2 (2.3) Pneumonia: 1 (1.1) Volume overload: 1 (1.1)	Median 1.3 (IQR 0)	-
**Cheng et al. [28]**	LA: 67	Transperitoneal: 42 (62.7) Retroperitoneal: 25 (37.3)	Hypertensive crisis: 8 (11.9) Organ injury requiring suture: 2 (3) Cava injury requiring suture: 1 (1.5) Bleeding: 1 (1.5) Suspecting spleen rupture requiring open exploration: 1 (1.5) Transfusion: 18 (26.9)	120 (range 100–200)	n.r.	3 (4.5)	220 (range 190–260)	Electrolyte imbalance: 1 (1.5) Fever: 1 (1.5) Pneumonia: 1 (1.5) Heart failure: 1 (1.5) Transfusion: 14 (20.9)	13 (range 8–21)	-
	RA: 48	Transperitoneal: 35 (72.9) Retroperitoneal: 13 (27.1)	Ventricular fibrillation: 1 (2.1) Hypertensive crisis: 2 (4.2) Organ injury requiring suture: 1 (2.1) Bleeding: 1 (2.1) Diaphragmatic rupture requiring suture: 1 (2.1) Transfusion: 18 (37.5)	50 (range 30–212.5)	n.r.	-	190 (range 170–215)	Electrolyte imbalance: 1 (2.1) Transfusion: 13 (27.1)	12.5 (range 8–18)	-
**Huang et al. [29]**	LA: 153	Retroperitoneal	Hemodynamic instability: 95 (62.1)	100 (range 50–200)	Greatest: 160 (range 145.5–175.5) Lowest: 94.6 ± 12.3	-	145 (range 115–190)	39 (25.5)	6 (range 5–7)	-
	RA: 68	Retroperitoneal	Hemodynamic instability: 29 (42.6)	100 (range 50–200)	Greatest: 161 (range 140.5–179.5) Lowest: 95.5 ± 12.7	-	150 (range 120–193.8)	13 (19.1)	6 (range 6–7)	-

EBL: estimated blood loss. SBP: systolic blood pressure. HR: heart rate. SD: standard deviation. LOS: length of hospital stay. TTYA: laparoscopic adrenalectomy. RA: robotic adrenalectomy. ^a^: two bilateral adrenalectomy. ^b^: one bilateral adrenalectomy. ^c^: 8 bilateral adrenalectomy. IQR: interquartile range. SEM: standard error of mean. n.r.: not reported.

## References

[1] Omura M., Saito J., Yamaguchi K., Kakuta Y., Nishikawa T. (2004). Prospective study on the prevalence of secondary hypertension among hypertensive patients visiting a general outpatient clinic in Japan. Hypertens. Res..

[2] Zelinka T., Eisenhofer G., Pacak K. (2007). Pheochromocytoma as a catecholamine producing tumor: Implications for clinical practice. Stress.

[3] Patel D., Phay J.E., Yen T.W.F., Dickson P.V., Wang T.S., Garcia R., Yang A.D., Solórzano C.C., Kim L.T. (2020). Update on Pheochromocytoma and Paraganglioma from the SSO Endocrine/Head and Neck Disease-Site Work Group. Part 1 of 2: Advances in Pathogenesis and Diagnosis of Pheochromocytoma and Paraganglioma. Ann. Surg. Oncol..

[4] De Crea C., Pennestri F., Voloudakis N., Sessa L., Procopio P.F., Gallucci P., Bellantone R., Raffaelli M. (2022). Robot-assisted vs laparoscopic lateral transabdominal adrenalectomy: A propensity score matching analysis. Surg. Endosc..

[5] Yu R., Nissen N.N., Bannykh S.I. (2012). Cardiac complications as initial manifestation of pheochromocytoma: Frequency, outcome, and predictors. Endocr. Pract..

[6] Bihain F., Klein M., Nomine-Criqui C., Brunaud L. (2020). Robotic adrenalectomy in patients with pheochromocytoma: A systematic review. Gland Surg..

[7] Li J., Wang Y., Chang X., Han Z. (2020). Laparoscopic adrenalectomy (LA) vs open adrenalectomy (OA) for pheochromocytoma (PHEO): A systematic review and meta-analysis. Eur. J. Surg. Oncol..

[8] Fu S.Q., Wang S.Y., Chen Q., Liu Y.T., Li Z.L., Sun T. (2020). Laparoscopic versus open surgery for pheochromocytoma: A meta-analysis. BMC Surg..

[9] Mihai I., Boicean A., Teodoru C.A., Grigore N., Iancu G.M., Dura H., Bratu D.G., Roman M.D., Mohor C.I., Todor S.B. (2023). Laparoscopic Adrenalectomy: Tailoring Approaches for the Optimal Resection of Adrenal Tumors. Diagnostics.

[10] Inaishi T., Kikumori T., Takeuchi D., Ishihara H., Miyajima N., Shibata M., Takano Y., Nakanishi K., Noda S., Kodera Y. (2018). Obesity Does Not Affect Peri- and Postoperative Outcomes of Transabdominal Laparoscopic Adrenalectomy. Nagoya J. Med. Sci..

[11] Wang L., Zeng W., Wu Y., Gong Z. (2024). Comparison of clinical efficacy and safety between robotic-assisted and laparoscopic adrenalectomy for pheochromocytoma: A systematic review and meta-analysis. J. Robot. Surg..

[12] Xia Z., Li J., Peng L., Yang X., Xu Y., Li X., Li Y., Zhang Z., Wu J. (2021). Comparison of Perioperative Outcomes of Robotic-Assisted vs Laparoscopic Adrenalectomy for Pheochromocytoma: A Meta-Analysis. Front. Oncol..

[13] Sforza S., Minervini A., Tellini R., Ji C., Bergamini C., Giordano A., Lu Q., Chen W., Zhang F., Ji H. (2021). Perioperative outcomes of robotic and laparoscopic adrenalectomy: A large international multicenter experience. Surg. Endosc..

[14] Park J.S., Lee K.Y., Kim J.K., Yoon D.S. (2009). The first laparoscopic resection of extra-adrenal pheochromocytoma using the da Vinci robotic system. J. Laparoendosc. Adv. Surg. Tech. A.

[15] Page M.J., McKenzie J.E., Bossuyt P.M., Boutron I., Hoffmann T.C., Mulrow C.D., Shamseer L., Tetzlaff J.M., Akl E.A., Brennan S.E. (2021). The PRISMA 2020 statement: An updated guideline for reporting systematic reviews. BMJ.

[16] Higgins J.P.T., Thomas J., Chandler J., Cumpston M., Li T., Page M.J., Welch V.A. (2022). Cochrane Handbook for Systematic Reviews of Interventions Version 6.3.

[17] Goossen K., Tenckhoff S., Probst P., Grummich K., Mihaljevic A.L., Büchler M.W., Diener M.K. (2018). Optimal literature search for systematic reviews in surgery. Langenbecks Arch. Surg..

[18] Sterne J.A., Hernán M.A., Reeves B.C., Savović J., Berkman N.D., Viswanathan M., Henry D., Altman D.G., Ansari M.T., Boutron I. (2016). ROBINS-I: A tool for assessing risk of bias in non-randomized studies of interventions. BMJ.

[19] Sterne J.A.C., Savović J., Page M.J., Elbers R.G., Blencowe N.S., Boutron I., Cates C.J., Cheng H.Y., Corbett M.S., Eldridge S.M. (2019). RoB 2: A revised tool for assessing risk of bias in randomised trials. BMJ.

[20] Hozo S.P., Djulbegovic B., Hozo I. (2005). Estimating the mean and variance from the median, range, and the size of a sample. BMC Med. Res. Methodol..

[21] DerSimonian R., Laird N. (2015). Meta-analysis in clinical trials revisited. Contemp. Clin. Trials.

[22] (2024). GRADEpro GDT: GRADEpro Guideline Development Tool [Software]. McMaster University and Evidence Prime.

[23] Aliyev S., Karabulut K., Agcaoglu O., Wolf K., Mitchell J., Siperstein A., Berber E. (2013). Robotic versus laparoscopic adrenalectomy for pheochromocytoma. Ann. Surg. Oncol..

[24] Ma W., Mao Y., Zhuo R., Dai J., Fang C., Wang C., Zhao J., He W., Zhu Y., Xu D. (2020). Surgical outcomes of a randomized controlled trial compared robotic versus laparoscopic adrenalectomy for pheochromocytoma. Eur. J. Surg. Oncol..

[25] Fu S.Q., Zhuang C.S., Yang X.R., Xie W.J., Gong B.B., Liu Y.F., Liu J., Sun T., Ma M. (2020). Comparison of robot-assisted retroperitoneal laparoscopic adrenalectomy versus retroperitoneal laparoscopic adrenalectomy for large pheochromocytoma: A single-centre retrospective study. BMC Surg..

[26] Fang A.M., Rosen J., Saidian A., Bae S., Tanno F.Y., Chambo J.L., Bloom J., Gordetsky J., Srougi V., Phillips J. (2020). Perioperative outcomes of laparoscopic, robotic, and open approaches to pheochromocytoma. J. Robot. Surg..

[27] Isiktas G., Nazli Avci S., Ergun O., Krishnamurthy V., Jin J., Siperstein A., Berber E. (2022). Laparoscopic versus robotic adrenalectomy in pheochromocytoma patients. J. Surg. Oncol..

[28] Cheng Y., Zhu Y. (2023). Comparison of Perioperative Outcomes Between Laparoscopic and Robot-Assisted Adrenalectomy for Large Pheochromocytoma (≥5 cm): A Retrospective Study. Cancer Manag. Res..

[29] Huang H., Sun T., Liu Z. (2024). Robot-assisted versus laparoscopic pheochromocytoma resection and construction of a nomogram to predict perioperative hemodynamic instability. Eur. J. Surg. Oncol..

[30] Giordano A., Alemanno G., Bergamini C., Valeri A., Prosperi P. (2021). Laparoscopic adrenalectomy for giant adrenal tumours: Technical considerations and surgical outcome. J. Minimal Access Surg..

[31] Öz B., Cücük Ö., Gök M., Akcan A., Sözüer E. (2024). Laparoscopic transperitoneal adrenalectomy for adrenal tumours of 6 cm or greater: A single-centre experience. J. Minimal Access Surg..

[32] Gagner M., Lacroix A., Bolté E. (1992). Laparoscopic adrenalectomy in Cushing’s syndrome and pheochromocytoma. N. Engl. J. Med..

[33] Giordano A., Feroci F., Podda M., Botteri E., Ortenzi M., Montori G., Guerrieri M., Vettoretto N., Agresta F., Bergamini C. (2023). Minimally invasive versus open adrenalectomy for adrenocortical carcinoma: The keys surgical factors influencing the outcomes-a collective overview. Langenbecks Arch. Surg..

[34] Ortenzi M., Balla A., Ghiselli R., Vergari R., Silecchia G., Guerrieri E., Maria Paganini A., Guerrieri M. (2019). Minimally invasive approach to the adrenal gland in obese patients with Cushing’s syndrome. Minim. Invasive Ther. Allied Technol..

[35] Verhoeff K., Parente A., Wang Y., Wang N., Wang Z., Śledziński M., Hellmann A., Raffaelli M., Pennestrì F., Sywak M. (2025). Outcomes for Patients with Obesity Undergoing Adrenalectomy for Pheochromocytoma: An International Multicenter Analysis. Ann. Surg. Oncol..

[36] Du L., Yang Z., Qi J., Wang Y. (2022). Robotic adrenalectomy versus laparoscopic adrenalectomy for pheochromocytoma: A systematic review and meta-analysis. Wideochir. Inne Tech. Maloinwazyjne.

[37] Balla A., Corallino D., Ortenzi M., Palmieri L., Meoli F., Guerrieri M., Paganini A.M. (2022). Cancer risk in adrenalectomy: Are adrenal lesions equal or more than 4 cm a contraindication for laparoscopy?. Surg. Endosc..

[38] Alzelfawi L., Almajed E., Alhindawi Z., AlDosari L., Alhumaidan A., Alharthi B. (2024). Feasibility of laparoscopic adrenalectomy in adrenal masses greater than 5 centimeters: A systematic review and meta-analysis. Gland Surg..

[39] Alberici L., Paganini A.M., Ricci C., Balla A., Ballarini Z., Ortenzi M., Casole G., Quaresima S., Di Dalmazi G., Ursi P. (2022). Development and validation of a preoperative “difficulty score” for laparoscopic transabdominal adrenalectomy: A multicenter retrospective study. Surg. Endosc..

[40] Zhang J., Hu K., Qing J., Chen J., Li C., Zhou Y. (2024). Hyper-realistic rendering-assisted laparoscopic adrenalectomy for giant adrenal tumors: A pilot study. World J. Urol..

[41] Gaillard M., Razafinimanana M., Challine A., Araujo R.L.C., Libé R., Sibony M., Barat M., Bertherat J., Dousset B., Fuks D. (2023). Laparoscopic or Open Adrenalectomy for Stage I–II Adrenocortical Carcinoma: A Retrospective Study. J. Clin. Med..

[42] Paganini A.M., Balla A., Guerrieri M., Lezoche G., Campagnacci R., D’Ambrosio G., Quaresima S., Antonica M.V., Lezoche E. (2014). Laparoscopic transperitoneal anterior adrenalectomy in pheochromocytoma: Experience in 62 patients. Surg. Endosc..

[43] Chai Y.J., Yu H.W., Song R.Y., Kim S.J., Choi J.Y., Lee K.E. (2019). Lateral transperitoneal adrenalectomy versus posterior retroperitoneoscopic adrenalectomy for benign adrenal gland disease: Randomized controlled trial at a single tertiary medical centre. Ann. Surg..

[44] Zhou Y., Tai Y., Shang J. (2025). Progress in treatment and follow-up of pheochromocytoma. Eur. J. Surg. Oncol..

[45] Habeeb T.A.A.M., Elias A.A., Adam A.A.M., Gadallah M.A., Ahmed S.M.A., Khyrallh A., Alsayed M.H., Awad E.T.K., Ibrahim E.A., Labib M.F. (2025). Early readmission after adrenalectomy for pheochromocytoma. A retrospective study. Langenbecks Arch. Surg..

